# Supplementary material for: CODEX2: full-spectrum copy number variation detection by high-throughput DNA sequencing
Source: Genome Biol. 2018 Nov 26;19:202. doi: 10.1186/s13059-018-1578-y (PMC6260772; doi:10.1186/s13059-018-1578-y)
Supplement: Supplementary file 1 — Algorithms S1-S3. Figures S1-S7. Table S4-S5, S7-S11. Supplementary materials. (PDF 1721 kb) [file 13059_2018_1578_MOESM1_ESM.pdf]

Algorithm S1. Parameter estimation algorithm for CODEX.

Initialization:

$\beta^{old} = 1^n$, $g = 0^{n\times K}$, $h = 0^{m\times K}$.

Iteration:

1. For each sample j, fit a smoothing spline $Y_{:j}/N_j\beta^{old}\exp(g \times h_{j:}^T) \sim GC$ to get $f_j(GC)$.
2. For each exon i, update β_i as $\beta_i^{new} = \text{median}\Big([Y/Nf(GC)\exp(g \times h^T)]_{i:}\Big)$.
3. Denote $Z = Nf(GC)\beta^{new}$. Apply SVD to row-centered $\log(Y/Z)$ to obtain the K right singular vectors and use as h^{old}.
   (a) For each i, fit Poisson log-linear regression with $Y_{i:}$ as response, h^{old} as covariates, $\log(Z_{i:})$ as fixed offset to obtain updated estimates as $\{g_{i1}, \cdots, g_{iK}\}$.
   (b) For each j, fit Poisson log-linear regression with $Y_{:j}$ as response, g as covariates, $\log(Z_{:j})$ as fixed offset to obtain updated estimates as $\{h_{j1}^{new}, \cdots, h_{jK}^{new}\}$.
   (c) Center each row of $g \times (h^{new})^T$ and apply SVD to the row-centered matrix to obtain the K right singular vectors to update h^{new}.
   (d) Repeat steps (a) to (c) with $h^{old} = h^{new}$ till convergence to obtain h, g.
4. Repeat steps 1 to 3 with $\beta^{old} = \beta^{new}$ till convergence.

Algorithm S2. Parameter estimation algorithm for CODEX2 with negative control samples.

Let J_c be the indices of the control samples.

1. Apply CODEX to the null samples $Y_{:J_c}$and GC using Supplementary Algorithm 1 to get an estimate of $h_{J_c:}, g, \beta$ and $f_{J_c}(GC)$.
2. For case sample $j \notin J_c$, initiate $h_{j1}^{old} = \cdots = h_{jK}^{old} = 0$.
   (a) Fit a smoothing spline $Y_{:j}/N_j\beta \exp(g \times h_{j:}^{old^T}) \sim GC$ to get $f_j(GC)$.
   (b) Denote $Z = Nf(GC)\beta$. Fit Poisson log-linear regression with $Y_{:j}$ as response, g as covariates, $\log(Z_{:j})$ as fixed offset to obtain updated estimates as $\{h_{j1}^{new}, \cdots, h_{jK}^{new}\}$.
   (c) Repeat steps (a) to (b) with $h_{j:}^{old} = h_{j:}^{new}$ till convergence to obtain $h_{j:}$, $f_j(GC)$.

Algorithm S3. Parameter estimation algorithm for CODEX2 without negative control samples.

Let I^* be the indices of exons with CNVs with high population frequencies.

1. Apply CODEX to the null regions $Y_{-I^*:}$and GC_{-I^*} using Supplementary Algorithm 1 to get an estimate of $h, g_{-I^*}, \beta_{-I^*}$ and $f(GC_{-I^*})$.
2. Use the estimated non-parametric smooth spline function to estimate the GC content bias in the non-null regions $f(GC_{I^*})$ with the corresponding GC_{I^*}.
3. For each $i^* \in I^*$, adopt an EM algorithm to estimate β_{i^*} and $\{g_{i^*1}, \cdots, g_{i^*K}\}$.
   (a) E-step:
$$\begin{aligned} p_1 &= P\big(Y_{i*j} \mid N_j, f_j(GC_{i^*}), Z_{i^*j} = 1, \hat{\mu}_{i^*}\big)\hat{\pi}_{i^*}, \\ p_0 &= P\big(Y_{i*j} \mid N_j, f_j(GC_{i^*}), Z_{i^*j} = 0, \hat{\mu}_{i^*}\big)(1 - \hat{\pi}_{i^*}), \\ \hat{Z}_{i^*j} &= p_1/(p_1 + p_0), \end{aligned}$$
   where $\hat{\mu}_{i^*}$ and $\hat{\pi}_{i^*}$ are from the previous M-step and $P(Y_{i^*j} \mid \lambda_{i^*j})$ is the probability for Poisson distribution with λ_{i^*j} calculated based on the given parameters.
   (b) M-step:
$$\hat{\pi}_{i^*} = \frac{1}{m}\sum_{j=1}^{m}\hat{Z}_{i^*j},$$
   where $\hat{Z}_{i^*j}$ is from the previous E-step. Run Poisson log-linear regression with $Y_{i^*:}$ as response, h and $\hat{Z}_{i^*:}$ as covariates, and $\log(N \times f(GC))_{i^*:}$ as fixed offsets to obtain estimates of $\hat{\beta}_{i^*}$ as intercept, $\{\hat{g}_{i^*1}, ..., \hat{g}_{i^*K}\}$ and $\hat{\mu}_{i^*}$ as coefficients.

Figure S1. WGS CNV calls from Phase 3 release by two callers. WGS CNV calls from Phase 3 release by two callers. Number of deletion and duplication calls are reported in the cohort of 90 HapMap samples that we used to benchmark. There is substantial discrepancy between the results by the two callers. These CNV calls are contaminated by false positives.

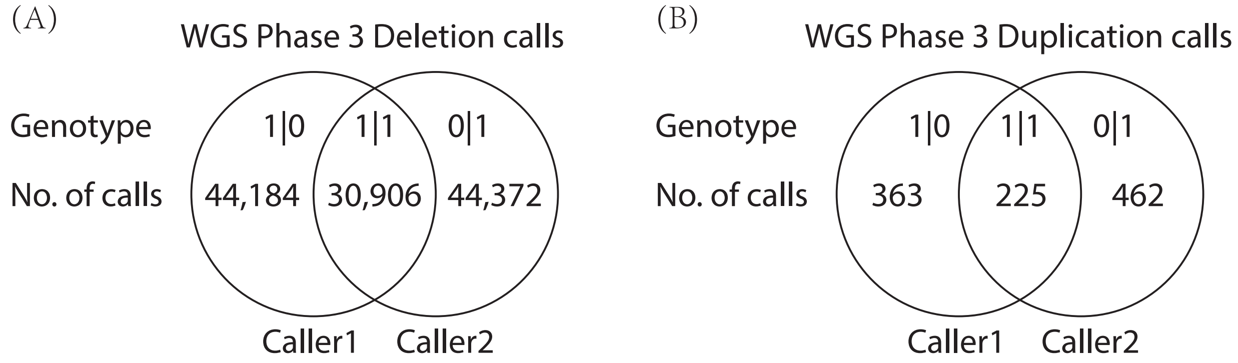

Figure S2. WGS CNV calls in trio dataset by CNVnator and CODEX2. WGS CNV calls in family trio dataset by (A) CNVnator and (B) CODEX2. CNVnator and CODEX2 return on average 1011 and 188 CNVs per individual, respectively. Results by CODEX2 show higher similarity between child and parents and lower similarity between non-related individuals.

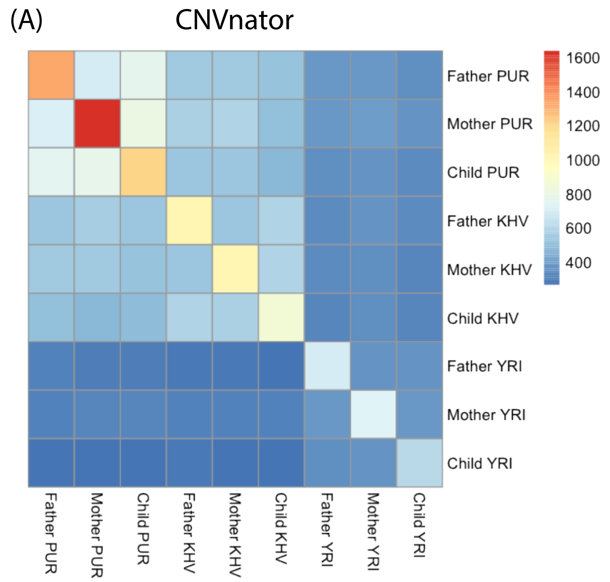

Figure S3. Performance assessment of WGS CNV calls. Performance assessment of WGS CNV calls by CNVnator and CODEX2. CNVs that are true positives are more likely to be shared between related individuals than between unrelated individuals. (A) Proportion of CNVs detected in mother that are also in father. Except for common CNVs that are shared between non-related individuals, this proportion should be low and is indicative of specificity. (B) Proportion of CNVs detected in child that are also in either parents. This Mendelian concordance should be high and is indicative of sensitivity. (C) Ratio of concordance between related individuals from (A) and unrelated individuals from (B) as a joint measurement of sensitivity and specificity.

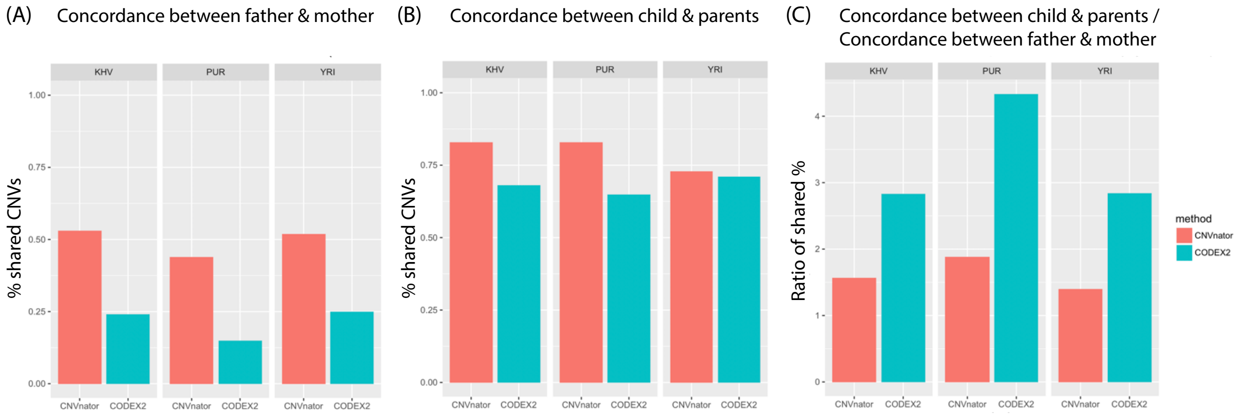

Figure S4. Model selection and parameter estimation in spike-in studies. Model selection and parameter estimation in spike-in studies. (A) Number of latent factors is determined by variance reduction and BIC based on the null read depth after quality control procedures and is kept the same throughout the spike-in studies. (B-D) True underlying parameters and estimated parameters by CODEX and CODEX2. CODEX biasedly estimates the exon-specific latent factor $\{g_1, g_2, g_3\}$ for the exons, where common CNV signals are *in silico* added.

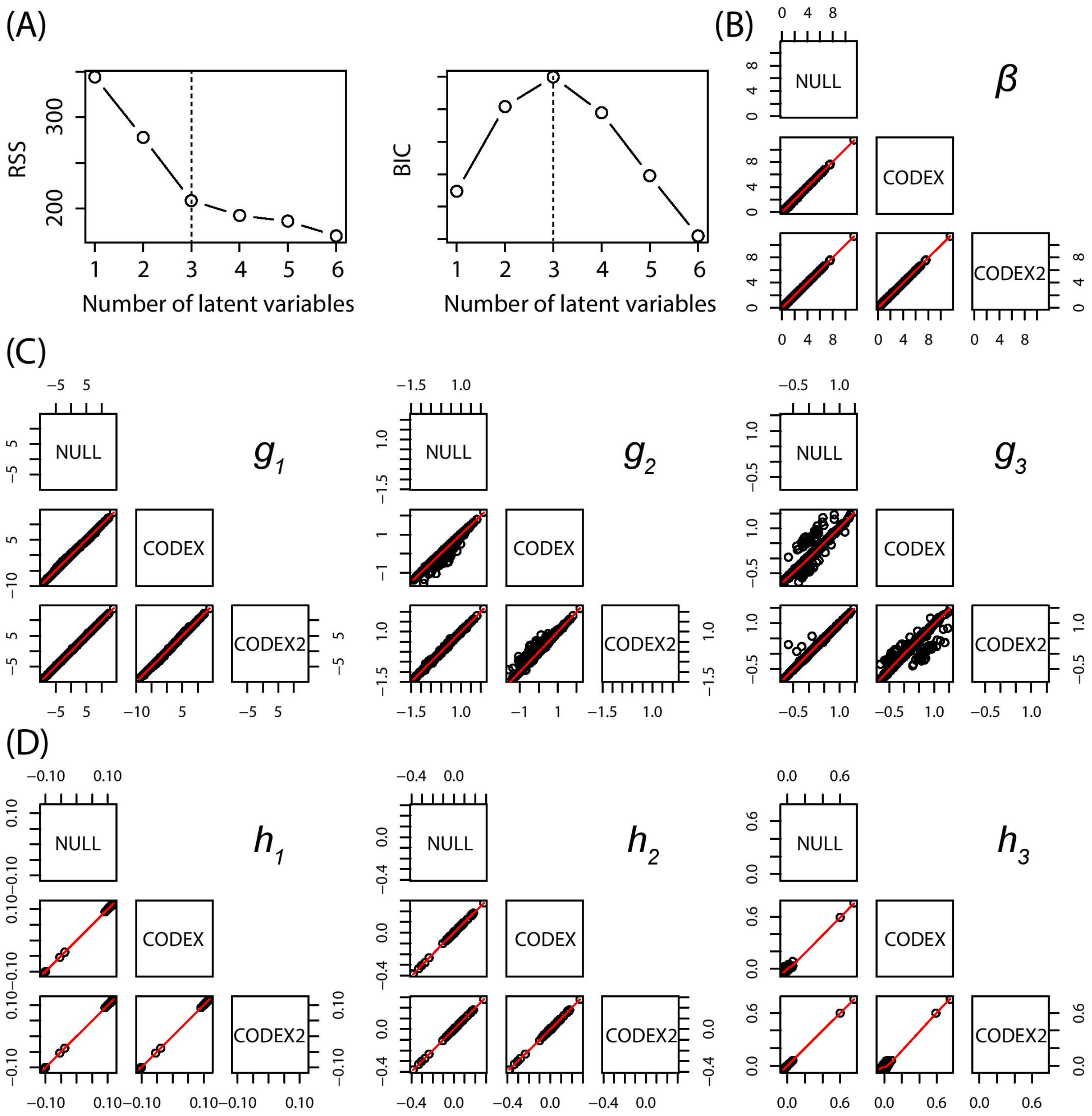

Figure S5. Biased parameter estimation by CODEX with common CNV signals. Parameter estimation by CODEX when the null data is mixed with alternatives with different frequencies. Here we focus on a specific exon i^* and try to estimate g_{i^*1} from Poisson generalized linear model taking h_1 as known from previous iteration.

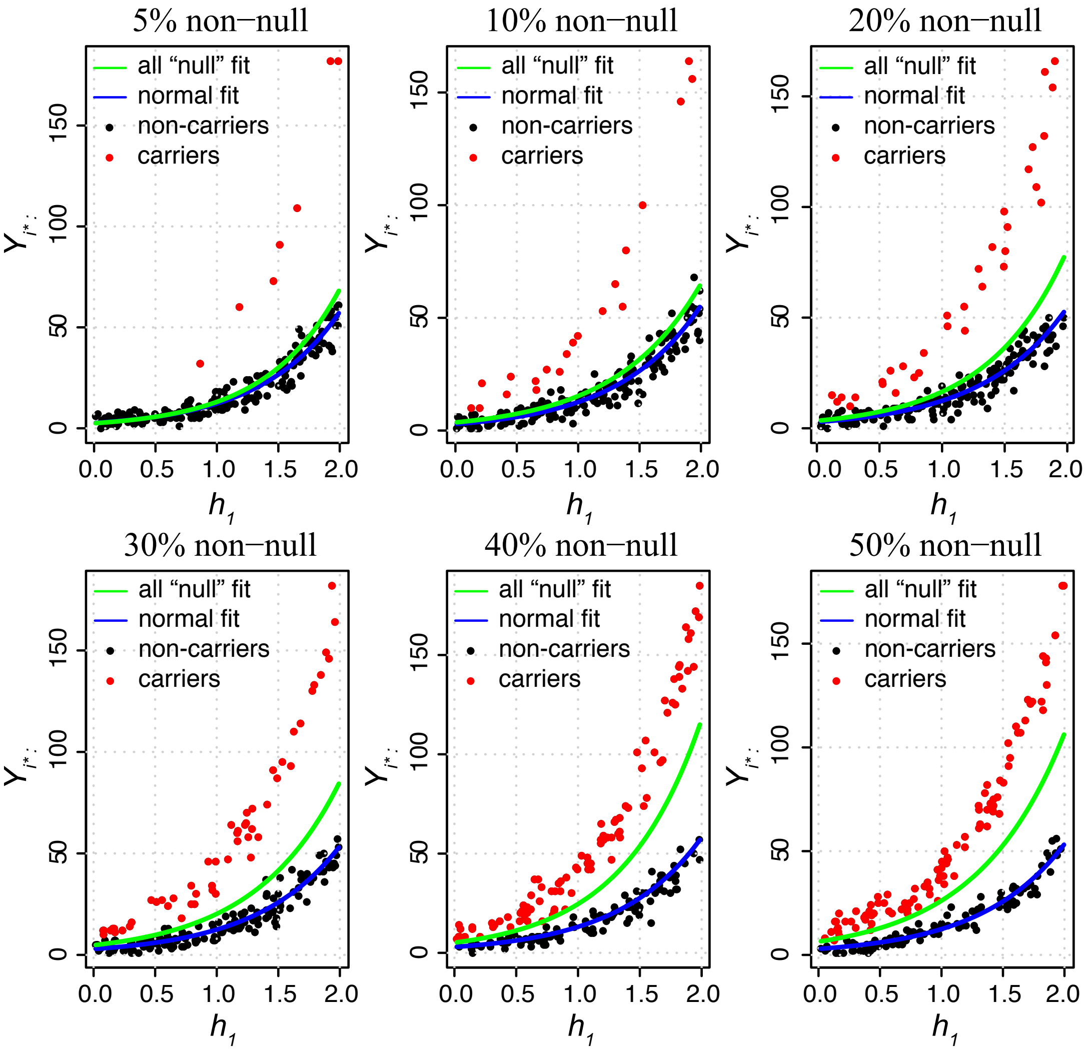

Figure S6. Assessment of precision and recall rates with different CNV lengths. Assessment of precision and recall rates with different CNV lengths. CODEX2 has nearly perfect performance compared to CODEX and SVD-based method, which suffer from low recall and precision rates for large and common CNV singals.

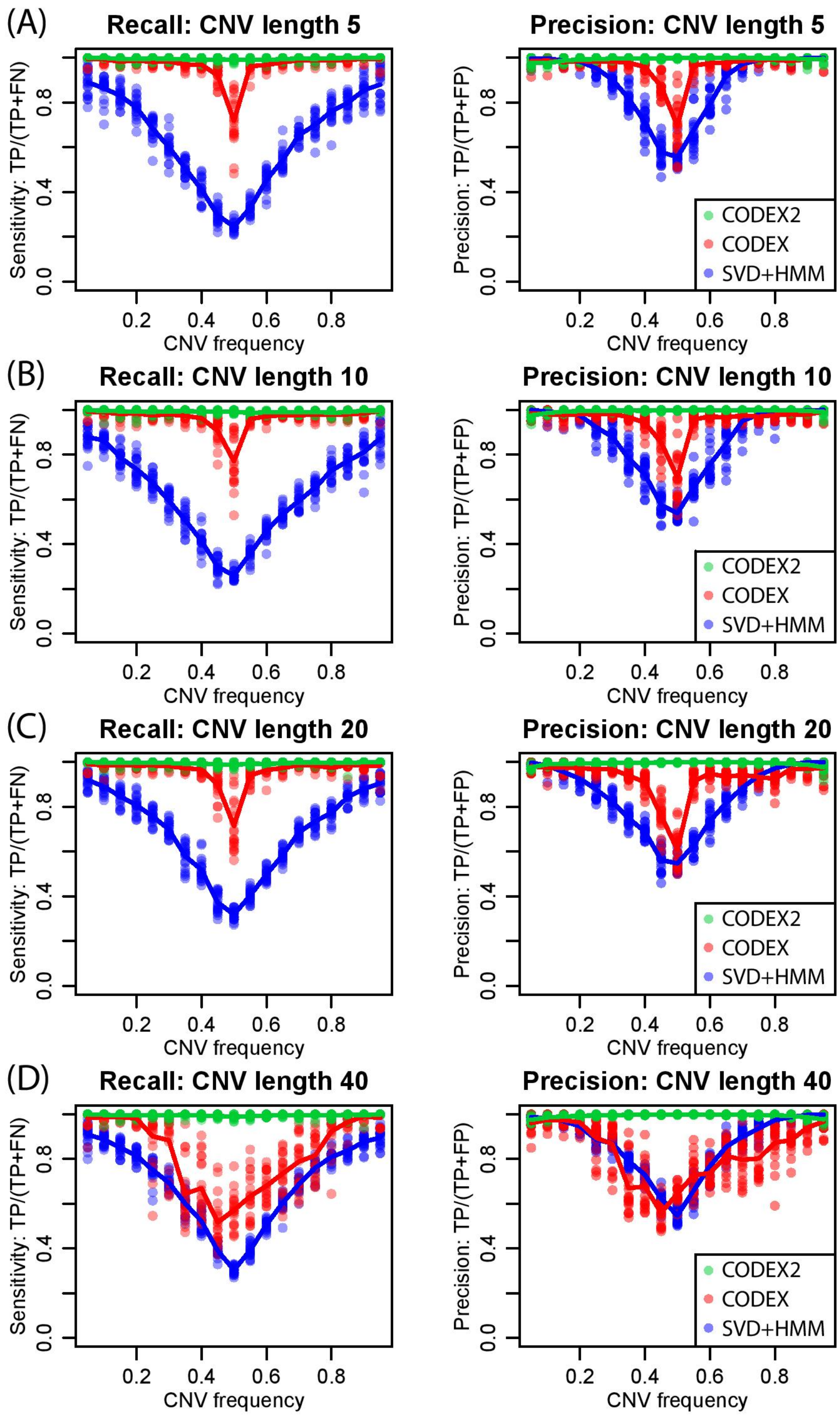

Figure S7. Relationship between CNV state and batch effect. Relationship between CNV state and batch effect. (A) The null read depth from the 1000 Genomes Project after quality control procedures show distinct batch effect between two centers, where the samples were sequenced. An example spike-in run where the CNVs are mostly added to the samples from one center. The CNV state and the batch effect are highly correlated. (B) An example of Poisson log-linear regression to estimate the exon-specific latent factor for a specific exon in a common CNV region. The vertical axis is Y (in this case the spike-in read depth) while the horizontal axis is the λ (estimated null read depth by CODEX and CODEX2). If there is no CNV, these two quantities should be approximately equal along the diagonal line. CODEX's fitted results separate the carriers and non-carriers at two sides of the diagonal line. CODEX2 adopts the EM algorithm with the non-carriers lying along the diagonal line as expected and the carriers below the diagonal line called as deletions. (C) Heatmap of the normalization results in all non-null exons across all samples. CODEX suffers from low sensitivity for detecting true deletions in carriers and false positives as deletions in non-carriers.

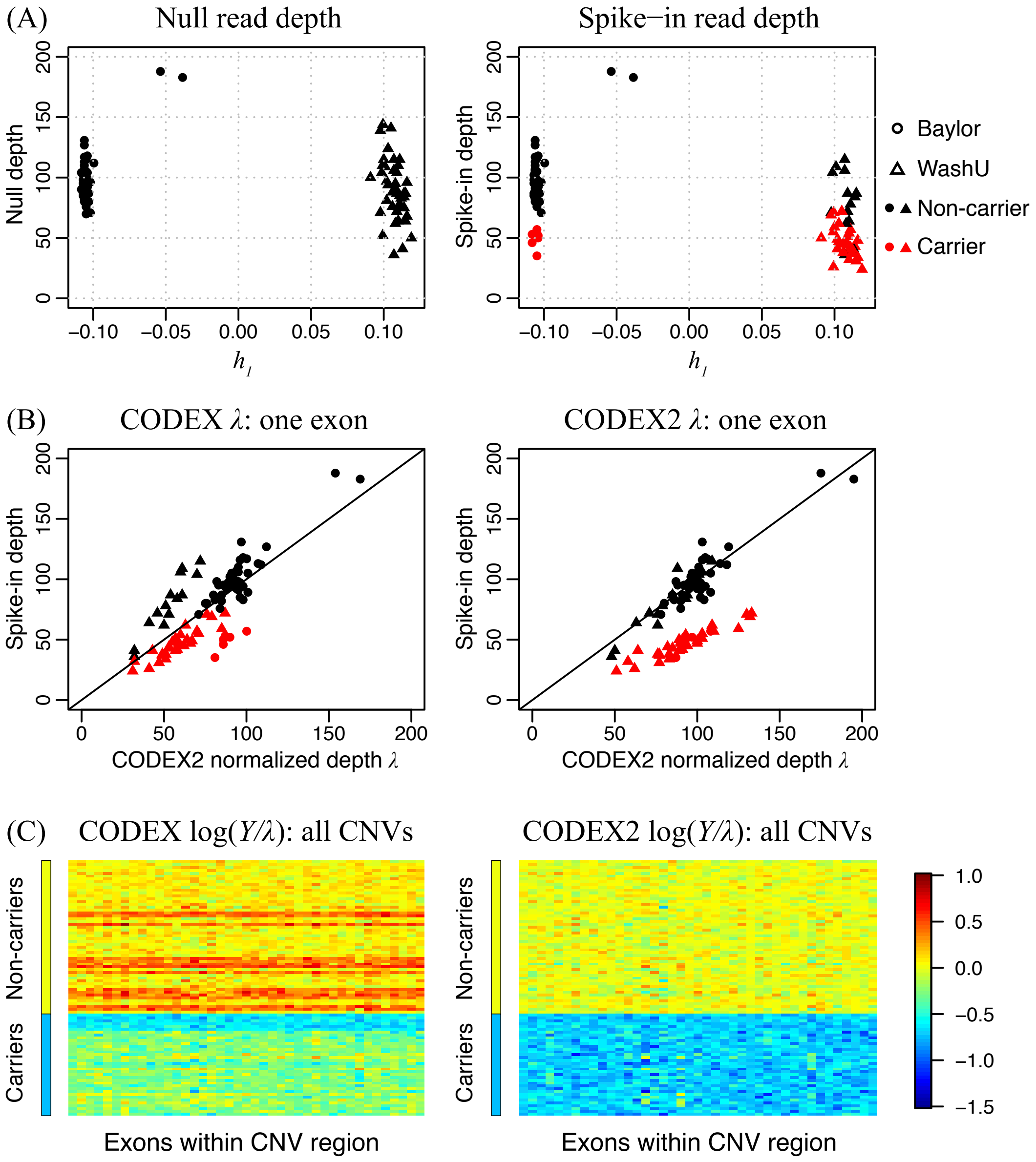

Table S4. Performance assessment and benchmark of WES CNV calls. Performance assessment and benchmark of WES CNV calls from the 1000 Genomes Project in the 90 HapMap samples by CODEX2, CODEX, CLAMMS, XHMM, and EXCAVATOR. F-measure is defined as harmonic mean of precision and recall.

HapMap3

	All								Common								Rare							
	TP	TN	FP	FN	Na	Sensitivity	Precision	F_measure	TP	TN	FP	FN	Na	Sensitivity	Precision	F_measure	TP	TN	FP	FN	Na	Sensitivity	Precision	F_measure
XHMM	56	2004	30	237	13	0.1911	0.6512	0.2955	24	839	2	203	12	0.1057	0.9231	0.1897	33	1246	28	42	1	0.44	0.541	0.4853
EXCAVATOR	47	1937	98	245	13	0.161	0.3241	0.2151	31	803	39	195	12	0.1372	0.4429	0.2095	16	1215	59	59	1	0.2133	0.2133	0.2133
CLAMMS	175	2027	7	118	13	0.5973	0.9615	0.7368	141	835	6	86	12	0.6211	0.9592	0.754	34	1273	1	41	1	0.4533	0.9714	0.6182
CODEX	132	1995	39	161	13	0.4505	0.7719	0.569	86	832	9	141	12	0.3789	0.9053	0.5342	46	1244	30	29	1	0.6133	0.6053	0.6093
CODEX2	272	1948	86	21	13	0.9283	0.7598	0.8356	218	821	20	9	12	0.9604	0.916	0.9376	63	1205	69	12	1	0.84	0.4773	0.6087

Conrad et al.

	All								Common								Rare							
	TP	TN	FP	FN	Na	Sensitivity	Precision	F_measure	TP	TN	FP	FN	Na	Sensitivity	Precision	F_measure	TP	TN	FP	FN	Na	Sensitivity	Precision	F_measure
XHMM	66	3401	14	481	88	0.1207	0.825	0.2105	41	1659	6	404	50	0.0921	0.8723	0.1667	27	1823	9	83	38	0.2455	0.75	0.3699
EXCAVATOR	59	3396	19	488	88	0.1079	0.7564	0.1888	52	1639	26	393	50	0.1169	0.6667	0.1989	11	1828	4	99	38	0.1	0.7333	0.176
CLAMMS	229	3403	13	317	88	0.4194	0.9463	0.5812	187	1654	11	258	50	0.4202	0.9444	0.5816	42	1828	4	68	38	0.3818	0.913	0.5385
CODEX	167	3351	64	380	88	0.3053	0.7229	0.4293	136	1605	59	310	50	0.3049	0.6974	0.4243	33	1827	5	77	38	0.3	0.8684	0.4459
CODEX2	332	3319	96	215	88	0.6069	0.7757	0.681	298	1591	74	147	50	0.6697	0.8011	0.7295	42	1808	24	68	38	0.3818	0.6364	0.4773

McCarroll et al.

	All								Common								Rare							
	TP	TN	FP	FN	Na	Sensitivity	Precision	F_measure	TP	TN	FP	FN	Na	Sensitivity	Precision	F_measure	TP	TN	FP	FN	Na	Sensitivity	Precision	F_measure
XHMM	86	2367	21	371	35	0.1882	0.8037	0.305	49	1122	10	340	9	0.126	0.8305	0.2188	42	1325	12	35	26	0.5455	0.7778	0.6412
EXCAVATOR	69	2330	60	386	35	0.1516	0.5349	0.2363	48	1093	41	339	9	0.124	0.5393	0.2017	24	1318	19	53	26	0.3117	0.5581	0.4
CLAMMS	208	2380	8	249	35	0.4551	0.963	0.6181	157	1127	5	232	9	0.4036	0.9691	0.5699	51	1334	3	26	26	0.6623	0.9444	0.7786
CODEX	164	2367	22	292	35	0.3596	0.8817	0.5109	116	1113	20	272	9	0.299	0.8529	0.4427	57	1334	3	20	26	0.7403	0.95	0.8321
CODEX2	361	2364	25	95	35	0.7917	0.9352	0.8575	308	1113	20	80	9	0.7938	0.939	0.8603	62	1330	7	15	26	0.8052	0.8986	0.8493

Phase 3 WGS

	All								Common								Rare							
	TP	TN	FP	FN	Na	Sensitivity	Precision	F_measure	TP	TN	FP	FN	Na	Sensitivity	Precision	F_measure	TP	TN	FP	FN	Na	Sensitivity	Precision	F_measure
XHMM	47	2451	23	359	0	0.1158	0.6714	0.1975	20	937	0	303	0	0.0619	1	0.1166	28	1676	23	73	0	0.2772	0.549	0.3684
EXCAVATOR	34	2390	84	372	0	0.0837	0.2881	0.1298	21	910	28	301	0	0.0652	0.4286	0.1132	13	1641	58	88	0	0.1287	0.1831	0.1512
CLAMMS	238	2472	2	168	0	0.5862	0.9917	0.7368	210	935	2	113	0	0.6502	0.9906	0.785	28	1699	0	73	0	0.2772	1	0.4341
CODEX	169	2377	104	230	0	0.4236	0.619	0.503	129	889	54	188	0	0.4069	0.7049	0.516	40	1650	50	60	0	0.4	0.4444	0.4211
CODEX2	269	2307	168	136	0	0.6642	0.6156	0.639	228	864	73	95	0	0.7059	0.7575	0.7308	52	1602	98	48	0	0.52	0.3467	0.416

Table S5. WGS family trio dataset from the 1000 Genomes Project. WGS family trio dataset from the 1000 Genomes Project. Mendelian concordance is used as metric to assess CNV calling quality.

Family_ID	Individual_ID	Relationship	Population	Bam_file
PR05	HG00731	father	PUR	HG00731.mapped.ILLUMINA.bwa.PUR.low_coverage.20130422.bam
PR05	HG00732	mother	PUR	HG00732.mapped.ILLUMINA.bwa.PUR.low_coverage.20130422.bam
PR05	HG00733	child	PUR	HG00733.mapped.ILLUMINA.bwa.PUR.low_coverage.20130415.bam
VN049	HG02026	father	KHV	HG02026.mapped.ILLUMINA.bwa.KHV.low_coverage.20130415.bam
VN049	HG02025	mother	KHV	HG02025.mapped.ILLUMINA.bwa.KHV.low_coverage.20130415.bam
VN049	HG02024	child	KHV	HG02024.mapped.ILLUMINA.bwa.KHV.low_coverage.20130415.bam
Y117	NA19239	father	YRI	NA19239.mapped.ILLUMINA.bwa.YRI.low_coverage.20130415.bam
Y117	NA19238	mother	YRI	NA19238.mapped.ILLUMINA.bwa.YRI.low_coverage.20130415.bam
Y117	NA19240	child	YRI	NA19240.mapped.ILLUMINA.bwa.YRI.low_coverage.20130415.bam

Table S7. Performance of CODEX and CODEX2 with different number of latent factors. Performance of CODEX and CODEX2 with different number of latent factors. CODEX and CODEX2 are applied to the melanoma targeted sequencing data set with the number of latent factors K ranging from 0 to 10. Correlations of the profiled losses and gains (r_{loss} and r_{gain} respectively) by CODEX and CODEX2 with those reported by TCGA, as well as the number of *BRAF* gains and *PTEN* losses out of 334 tumor samples are used as measurements. CNV profiles by CODEX2 are consistent since only the negative control samples (16 normal samples) are used to estimate the target-specific bias and artifacts. For CODEX, true CNV signals are attenuated with a large K resulting in less CNV events and lower correlations.

	**CODEX2**				**CODEX**			
K	r_{loss}	r_{gain}	*BRAF* gains	*PTEN* losses	r_{loss}	r_{gain}	*BRAF* gains	*PTEN* losses
0	0.787	0.859	288	256	0.571	0.112	170	146
1	0.840	0.841	297	230	0.589	-0.039	163	138
2	0.837	0.839	296	228	0.580	-0.003	161	133
3	0.841	0.830	296	227	0.567	0.013	160	117
4	0.837	0.839	292	229	0.581	0.034	158	113
5	0.842	0.848	302	229	0.556	0.046	158	110
6	0.845	0.845	301	229	0.546	0.033	155	108
7	0.842	0.853	298	231	0.520	0.049	142	107
8	0.838	0.859	295	237	0.453	0.027	130	97
9	0.831	0.858	299	234	0.435	0.042	128	95
10	0.835	0.859	297	237	0.442	0.039	125	98

Table S8. Recall and precision rates from simulations with deletions of different lengths. Recall rates (A) and precision rates (B) from simulations with deletions of different lengths (number of exons). Confidence interval (CI) is computed using the 5th and 95th percentile from 20 simulation runs.

(A)

		CODEX.CBS			CODEX2ns.CBS			CODEX2nr.CBS			SVD.HMM		
CNVfreq	CNVlength	median	sd	CI	median	sd	CI	median	sd	CI	median	sd	CI
0.10	5	1.00	0.00	(0.999,1)	1.00	0.00	(0.999,1)	1.00	0.00	(0.999,1)	1.00	0.04	(0.926,1)
0.10	10	1.00	0.01	(0.989,1)	1.00	0.00	(0.989,1)	1.00	0.01	(0.989,1)	0.92	0.05	(0.793,0.955)
0.10	20	1.00	0.00	(0.994,1)	1.00	0.00	(1,1)	1.00	0.00	(1,1)	0.95	0.04	(0.893,1)
0.10	40	1.00	0.00	(1,1)	1.00	0.00	(1,1)	1.00	0.00	(1,1)	0.99	0.03	(0.922,1)
0.20	5	1.00	0.05	(0.973,1)	1.00	0.00	(0.992,1)	1.00	0.00	(0.992,1)	0.92	0.08	(0.758,0.983)
0.20	10	1.00	0.00	(0.989,1)	1.00	0.00	(0.995,1)	1.00	0.00	(0.995,1)	0.86	0.05	(0.773,0.9)
0.20	20	1.00	0.00	(0.997,1)	1.00	0.00	(1,1)	1.00	0.00	(1,1)	0.93	0.03	(0.87,0.961)
0.20	40	1.00	0.04	(0.988,1)	1.00	0.00	(0.999,1)	1.00	0.00	(1,1)	0.98	0.05	(0.903,0.997)
0.30	5	1.00	0.01	(0.977,1)	1.00	0.00	(0.995,1)	1.00	0.00	(0.995,1)	0.82	0.06	(0.725,0.907)
0.30	10	0.99	0.01	(0.976,0.997)	1.00	0.00	(0.993,1)	1.00	0.00	(0.986,1)	0.73	0.06	(0.647,0.798)
0.30	20	1.00	0.00	(0.998,1)	1.00	0.00	(1,1)	1.00	0.00	(1,1)	0.88	0.03	(0.84,0.936)
0.30	40	1.00	0.04	(0.984,1)	1.00	0.00	(0.999,1)	1.00	0.00	(0.999,1)	0.93	0.06	(0.812,0.977)
0.40	5	0.99	0.02	(0.946,1)	1.00	0.00	(1,1)	1.00	0.00	(1,1)	0.61	0.07	(0.501,0.663)
0.40	10	0.96	0.02	(0.938,0.984)	1.00	0.00	(0.995,1)	1.00	0.00	(0.995,1)	0.54	0.06	(0.46,0.618)
0.40	20	1.00	0.01	(0.966,1)	1.00	0.00	(0.999,1)	1.00	0.00	(0.999,1)	0.78	0.03	(0.717,0.796)
0.40	40	0.94	0.46	(0,0.999)	1.00	0.00	(0.999,1)	1.00	0.00	(0.998,1)	0.81	0.41	(0,0.882)
0.50	5	0.79	0.07	(0.648,0.887)	1.00	0.00	(0.997,1)	1.00	0.08	(0.846,1)	0.30	0.05	(0.231,0.38)
0.50	10	0.85	0.06	(0.71,0.91)	1.00	0.00	(0.996,1)	1.00	0.08	(0.813,1)	0.28	0.04	(0.236,0.343)
0.50	20	0.90	0.06	(0.842,0.983)	1.00	0.00	(0.998,1)	1.00	0.00	(1,1)	0.64	0.04	(0.578,0.686)
0.50	40	0.90	0.28	(0.036,0.989)	1.00	0.00	(0.999,1)	1.00	0.04	(0.906,1)	0.74	0.23	(0.009,0.781)
0.60	5	1.00	0.00	(0.991,1)	1.00	0.00	(0.997,1)	1.00	0.00	(1,1)	0.57	0.07	(0.468,0.676)
0.60	10	0.98	0.04	(0.878,0.99)	1.00	0.00	(0.991,1)	0.99	0.01	(0.959,0.995)	0.54	0.05	(0.496,0.611)
0.60	20	1.00	0.02	(0.951,1)	1.00	0.00	(0.999,1)	1.00	0.01	(0.981,1)	0.73	0.04	(0.691,0.814)
0.60	40	0.00	0.19	(0,0.09)	1.00	0.00	(0.999,1)	1.00	0.00	(0.999,1)	0.00	0.14	(0,0.069)
0.70	5	1.00	0.01	(0.988,1)	1.00	0.00	(0.995,1)	1.00	0.00	(0.995,1)	0.81	0.05	(0.745,0.879)
0.70	10	0.99	0.01	(0.973,0.993)	1.00	0.00	(0.993,1)	0.99	0.01	(0.98,0.997)	0.74	0.04	(0.656,0.781)
0.70	20	1.00	0.00	(0.993,1)	1.00	0.00	(0.999,1)	1.00	0.00	(0.998,1)	0.87	0.03	(0.817,0.914)
0.70	40	0.04	0.48	(0,0.999)	1.00	0.00	(0.999,1)	1.00	0.00	(0.999,1)	0.03	0.45	(0,0.959)
0.80	5	1.00	0.00	(0.992,1)	1.00	0.00	(0.994,1)	1.00	0.00	(1,1)	0.91	0.06	(0.776,0.976)
0.80	10	0.99	0.01	(0.985,1)	1.00	0.00	(0.992,1)	0.99	0.01	(0.98,1)	0.80	0.05	(0.737,0.894)
0.80	20	1.00	0.01	(0.992,1)	1.00	0.00	(0.999,1)	1.00	0.00	(0.997,1)	0.92	0.04	(0.831,0.963)
0.80	40	1.00	0.01	(0.994,1)	1.00	0.00	(0.999,1)	1.00	0.00	(0.999,1)	0.97	0.03	(0.919,0.996)
0.90	5	1.00	0.00	(1,1)	1.00	0.00	(0.993,1)	1.00	0.00	(0.999,1)	0.97	0.04	(0.887,1)
0.90	10	1.00	0.01	(0.98,1)	1.00	0.00	(0.991,1)	1.00	0.01	(0.988,1)	0.89	0.04	(0.814,0.921)
0.90	20	1.00	0.00	(0.995,1)	1.00	0.00	(0.999,1)	1.00	0.00	(0.995,1)	0.99	0.03	(0.935,1)
0.90	40	1.00	0.00	(1,1)	1.00	0.00	(0.998,1)	1.00	0.00	(1,1)	0.99	0.01	(0.969,1)

(B)

		CODEX.CBS			CODEX2ns.CBS			CODEX2nr.CBS			SVD.HMM		
CNVfreq	CNVlength	median	sd	CI	median	sd	CI	median	sd	CI	median	sd	CI
0.10	5	1.00	0.00	(1,1)	1.00	0.00	(1,1)	1.00	0.00	(1,1)	1.00	0.00	(1,1)
0.10	10	1.00	0.00	(1,1)	1.00	0.00	(1,1)	1.00	0.00	(1,1)	1.00	0.00	(1,1)
0.10	20	1.00	0.00	(1,1)	1.00	0.00	(1,1)	1.00	0.00	(1,1)	1.00	0.00	(1,1)
0.10	40	1.00	0.00	(1,1)	1.00	0.00	(1,1)	1.00	0.00	(1,1)	1.00	0.01	(0.998,1)
0.20	5	1.00	0.02	(0.997,1)	1.00	0.00	(1,1)	1.00	0.00	(1,1)	1.00	0.01	(0.997,1)
0.20	10	1.00	0.01	(0.997,1)	1.00	0.00	(1,1)	1.00	0.00	(1,1)	1.00	0.02	(0.997,1)
0.20	20	1.00	0.00	(1,1)	1.00	0.00	(1,1)	1.00	0.00	(1,1)	1.00	0.00	(1,1)
0.20	40	1.00	0.03	(0.96,1)	1.00	0.00	(1,1)	1.00	0.00	(1,1)	1.00	0.02	(0.996,1)
0.30	5	1.00	0.01	(0.998,1)	1.00	0.00	(1,1)	1.00	0.00	(1,1)	1.00	0.01	(0.997,1)
0.30	10	1.00	0.01	(0.998,1)	1.00	0.00	(1,1)	1.00	0.00	(1,1)	1.00	0.01	(0.964,1)
0.30	20	1.00	0.00	(1,1)	1.00	0.00	(1,1)	1.00	0.00	(1,1)	1.00	0.00	(1,1)
0.30	40	1.00	0.02	(0.941,1)	1.00	0.00	(1,1)	1.00	0.00	(1,1)	1.00	0.02	(0.956,1)
0.40	5	1.00	0.02	(0.946,1)	1.00	0.00	(1,1)	1.00	0.00	(1,1)	0.99	0.04	(0.898,1)
0.40	10	1.00	0.01	(0.97,1)	1.00	0.00	(1,1)	1.00	0.00	(1,1)	0.98	0.02	(0.935,1)
0.40	20	0.98	0.02	(0.927,1)	1.00	0.00	(1,1)	1.00	0.00	(1,1)	1.00	0.03	(0.926,1)
0.40	40	0.92	0.23	(0.332,0.993)	1.00	0.00	(1,1)	1.00	0.00	(1,1)	0.95	0.31	(0.15,1)
0.50	5	0.62	0.06	(0.531,0.713)	1.00	0.00	(1,1)	1.00	0.09	(0.787,1)	0.56	0.07	(0.513,0.73)
0.50	10	0.63	0.04	(0.57,0.679)	1.00	0.00	(1,1)	1.00	0.08	(0.901,1)	0.57	0.05	(0.502,0.645)
0.50	20	0.55	0.03	(0.534,0.588)	1.00	0.00	(1,1)	1.00	0.00	(0.996,1)	0.51	0.02	(0.503,0.556)
0.50	40	0.55	0.09	(0.44,0.666)	1.00	0.00	(1,1)	1.00	0.09	(0.805,1)	0.54	0.09	(0.485,0.602)
0.60	5	1.00	0.00	(1,1)	1.00	0.00	(1,1)	1.00	0.00	(1,1)	1.00	0.03	(0.945,1)
0.60	10	1.00	0.02	(0.96,1)	1.00	0.00	(1,1)	1.00	0.01	(0.989,1)	0.94	0.05	(0.842,1)
0.60	20	0.97	0.05	(0.882,0.995)	1.00	0.00	(1,1)	1.00	0.01	(0.988,1)	0.91	0.06	(0.799,0.979)
0.60	40	0.08	0.24	(0,0.551)	1.00	0.00	(1,1)	1.00	0.00	(0.999,1)	1.00	0.18	(0.572,1)
0.70	5	1.00	0.01	(0.998,1)	1.00	0.00	(1,1)	1.00	0.00	(1,1)	1.00	0.02	(0.958,1)
0.70	10	1.00	0.00	(0.999,1)	1.00	0.00	(1,1)	1.00	0.00	(1,1)	1.00	0.01	(0.974,1)
0.70	20	0.99	0.02	(0.971,1)	1.00	0.00	(1,1)	1.00	0.00	(0.996,1)	0.98	0.04	(0.905,1)
0.70	40	0.49	0.39	(0,0.995)	1.00	0.00	(1,1)	1.00	0.01	(0.994,1)	0.98	0.16	(0.732,1)
0.80	5	1.00	0.00	(1,1)	1.00	0.00	(1,1)	1.00	0.00	(1,1)	1.00	0.01	(0.997,1)
0.80	10	1.00	0.01	(0.998,1)	1.00	0.00	(1,1)	1.00	0.00	(1,1)	1.00	0.00	(1,1)
0.80	20	1.00	0.04	(0.902,1)	1.00	0.00	(1,1)	1.00	0.00	(0.992,1)	0.99	0.03	(0.924,1)
0.80	40	0.99	0.09	(0.767,1)	1.00	0.00	(1,1)	1.00	0.00	(1,1)	1.00	0.02	(0.963,1)
0.90	5	1.00	0.00	(1,1)	1.00	0.00	(1,1)	1.00	0.00	(1,1)	1.00	0.00	(1,1)
0.90	10	1.00	0.00	(1,1)	1.00	0.00	(1,1)	1.00	0.00	(1,1)	1.00	0.00	(1,1)
0.90	20	1.00	0.02	(0.976,1)	1.00	0.00	(1,1)	1.00	0.00	(0.99,1)	0.99	0.01	(0.978,1)
0.90	40	1.00	0.06	(0.89,1)	1.00	0.00	(1,1)	1.00	0.00	(0.995,1)	1.00	0.01	(0.985,1)

Table S9. Recall and precision rates from simulations with copy number gains. Recall rates (A) and precision rates (B) from simulations with copy number gains. Confidence interval (CI) is computed using the 5th and 95th percentile from 20 simulation runs.

(A)

		CODEX.CBS			CODEX2ns.CBS			CODEX2nr.CBS			SVD.HMM		
CNVfreq	CNVlength	median	sd	CI	median	sd	CI	median	sd	CI	median	sd	CI
0.10	20	0.99	0.01	(0.986,1)	0.99	0.00	(0.988,1)	0.99	0.00	(0.988,1)	0.94	0.05	(0.827,0.988)
0.20	20	0.99	0.03	(0.962,0.997)	1.00	0.00	(0.989,1)	0.99	0.00	(0.989,1)	0.87	0.03	(0.834,0.937)
0.30	20	0.97	0.02	(0.92,0.987)	1.00	0.00	(0.993,0.998)	1.00	0.01	(0.976,0.998)	0.80	0.05	(0.723,0.873)
0.40	20	0.92	0.04	(0.853,0.956)	1.00	0.00	(0.993,0.999)	1.00	0.01	(0.985,0.997)	0.72	0.05	(0.641,0.787)
0.50	20	0.44	0.07	(0.31,0.52)	1.00	0.00	(0.984,0.998)	0.90	0.09	(0.735,0.989)	0.24	0.10	(0.137,0.405)
0.60	20	0.17	0.04	(0.124,0.227)	1.00	0.00	(0.988,0.997)	0.76	0.08	(0.626,0.877)	0.63	0.08	(0.458,0.685)
0.70	20	0.50	0.07	(0.398,0.586)	1.00	0.00	(0.988,0.997)	0.91	0.08	(0.789,0.959)	0.85	0.03	(0.811,0.892)
0.80	20	0.72	0.08	(0.615,0.829)	0.99	0.00	(0.986,0.998)	0.93	0.07	(0.8,0.974)	0.91	0.06	(0.775,0.966)
0.90	20	0.88	0.05	(0.788,0.928)	0.99	0.01	(0.978,0.998)	0.93	0.05	(0.829,0.989)	0.98	0.03	(0.92,0.995)

(B)

		CODEX.CBS			CODEX2ns.CBS			CODEX2nr.CBS			SVD.HMM		
CNVfreq	CNVlength	median	sd	CI	median	sd	CI	median	sd	CI	median	sd	CI
0.10	20	1.00	0.00	(1,1)	1.00	0.00	(1,1)	1.00	0.00	(1,1)	1.00	0.00	(1,1)
0.20	20	1.00	0.00	(1,1)	1.00	0.00	(1,1)	1.00	0.00	(1,1)	1.00	0.00	(1,1)
0.30	20	1.00	0.00	(1,1)	1.00	0.00	(1,1)	1.00	0.00	(1,1)	1.00	0.00	(1,1)
0.40	20	1.00	0.00	(1,1)	1.00	0.00	(1,1)	1.00	0.00	(1,1)	1.00	0.00	(1,1)
0.50	20	1.00	0.01	(0.982,1)	1.00	0.00	(1,1)	0.98	0.05	(0.862,1)	1.00	0.06	(0.911,1)
0.60	20	0.81	0.08	(0.647,0.881)	1.00	0.00	(1,1)	0.90	0.08	(0.758,0.99)	1.00	0.02	(0.957,1)
0.70	20	0.99	0.01	(0.969,1)	1.00	0.00	(1,1)	0.99	0.07	(0.845,1)	1.00	0.00	(0.998,1)
0.80	20	1.00	0.01	(0.986,1)	1.00	0.00	(1,1)	1.00	0.07	(0.786,1)	1.00	0.00	(0.997,1)
0.90	20	1.00	0.00	(1,1)	1.00	0.00	(1,1)	1.00	0.03	(0.938,1)	1.00	0.00	(0.995,1)

Table S10. Recall and precision rates for CNV detection in heterogeneous samples. Recall rates (A) and precision rates (B) from simulations with deletions in heterogeneous samples. Purity, p, refers to the proportion of cells having the deletion in a heterogeneous sample. For copy number loss, we spike in signals corresponding to copy number $p \times c + (1 - p) \times 2$, where c is sampled from a Gaussian distribution with mean 1. Confidence interval (CI) is computed using the 5th and 95th percentile from 20 simulation runs.

(A)

			CODEX.CBS			CODEX2ns.CBS			CODEX2nr.CBS			SVD.HMM		
purity	CNVfreq	CNVlength	median	sd	CI	median	sd	CI	median	sd	CI	median	sd	CI
0.90	0.10	20	1.00	0.00	(1,1)	1.00	0.00	(1,1)	1.00	0.00	(1,1)	0.98	0.04	(0.874,0.994)
0.90	0.20	20	1.00	0.00	(0.997,1)	1.00	0.00	(1,1)	1.00	0.00	(0.997,1)	0.90	0.03	(0.837,0.927)
0.90	0.40	20	1.00	0.00	(0.997,1)	1.00	0.00	(0.999,1)	1.00	0.00	(0.999,1)	0.71	0.03	(0.663,0.744)
0.70	0.10	20	1.00	0.01	(0.988,1)	1.00	0.01	(0.988,1)	1.00	0.01	(0.988,1)	0.82	0.08	(0.722,0.936)
0.70	0.20	20	1.00	0.00	(0.992,1)	1.00	0.00	(0.994,1)	1.00	0.00	(0.994,1)	0.75	0.06	(0.608,0.785)
0.70	0.40	20	0.99	0.01	(0.98,0.999)	1.00	0.00	(0.997,1)	1.00	0.00	(0.993,1)	0.46	0.03	(0.429,0.522)
0.50	0.10	20	0.99	0.02	(0.946,1)	0.99	0.01	(0.969,1)	0.99	0.01	(0.97,1)	0.52	0.09	(0.45,0.698)
0.50	0.20	20	0.99	0.01	(0.961,0.994)	1.00	0.01	(0.972,1)	0.99	0.01	(0.964,0.997)	0.40	0.04	(0.327,0.454)
0.50	0.40	20	0.93	0.05	(0.807,0.962)	0.99	0.01	(0.962,0.996)	0.95	0.06	(0.814,0.981)	0.17	0.03	(0.132,0.213)
0.30	0.10	20	0.74	0.12	(0.589,0.971)	0.85	0.10	(0.704,0.989)	0.82	0.12	(0.618,0.982)	0.14	0.04	(0.077,0.19)
0.30	0.20	20	0.62	0.11	(0.479,0.793)	0.88	0.07	(0.695,0.919)	0.68	0.09	(0.546,0.819)	0.08	0.03	(0.041,0.129)
0.30	0.40	20	0.29	0.06	(0.219,0.402)	0.88	0.04	(0.804,0.928)	0.38	0.05	(0.311,0.486)	0.03	0.01	(0.016,0.042)
0.10	0.10	20	0.00	0.00	(0,0)	0.00	0.00	(0,0)	0.00	0.03	(0,0.079)	0.00	0.00	(0,0)
0.10	0.20	20	0.00	0.00	(0,0)	0.00	0.03	(0,0.061)	0.00	0.01	(0,0.036)	0.00	0.00	(0,0)
0.10	0.40	20	0.00	0.00	(0,0)	0.01	0.02	(0,0.058)	0.00	0.00	(0,0)	0.00	0.00	(0,0)

(B)

			CODEX.CBS			CODEX2ns.CBS			CODEX2nr.CBS			SVD.HMM		
purity	CNVfreq	CNVlength	median	sd	CI	median	sd	CI	median	sd	CI	median	sd	CI
0.90	0.10	20	1.00	0.04	(0.991,1)	1.00	0.00	(1,1)	1.00	0.00	(1,1)	1.00	0.01	(0.984,1)
0.90	0.20	20	1.00	0.02	(0.995,1)	1.00	0.01	(0.997,1)	1.00	0.00	(1,1)	0.92	0.02	(0.868,0.938)
0.90	0.40	20	0.62	0.05	(0.558,0.7)	1.00	0.00	(1,1)	1.00	0.00	(1,1)	0.62	0.04	(0.544,0.647)
0.70	0.10	20	1.00	0.00	(1,1)	1.00	0.00	(1,1)	1.00	0.00	(1,1)	1.00	0.01	(0.984,1)
0.70	0.20	20	1.00	0.02	(0.953,1)	1.00	0.01	(0.997,1)	1.00	0.00	(1,1)	0.98	0.01	(0.961,0.996)
0.70	0.40	20	0.74	0.05	(0.662,0.823)	1.00	0.00	(1,1)	0.92	0.03	(0.874,0.958)	0.70	0.03	(0.665,0.755)
0.50	0.10	20	1.00	0.05	(0.885,1)	1.00	0.00	(1,1)	1.00	0.03	(0.994,1)	1.00	0.01	(0.978,1)
0.50	0.20	20	1.00	0.02	(0.944,1)	1.00	0.00	(1,1)	1.00	0.02	(0.953,1)	1.00	0.02	(0.982,1)
0.50	0.40	20	0.88	0.04	(0.823,0.92)	1.00	0.01	(0.999,1)	0.91	0.03	(0.875,0.956)	0.75	0.04	(0.705,0.798)
0.30	0.10	20	1.00	0.00	(1,1)	1.00	0.00	(1,1)	1.00	0.00	(1,1)	1.00	0.00	(1,1)
0.30	0.20	20	1.00	0.00	(1,1)	1.00	0.01	(0.997,1)	1.00	0.00	(1,1)	1.00	0.00	(1,1)
0.30	0.40	20	0.93	0.06	(0.815,1)	1.00	0.00	(1,1)	0.94	0.03	(0.926,1)	0.95	0.05	(0.854,1)
0.10	0.10	20			(NA,NA)			(NA,NA)	1.00	0.00	(1,1)			(NA,NA)
0.10	0.20	20			(NA,NA)	1.00	0.00	(1,1)	1.00	0.00	(1,1)			(NA,NA)
0.10	0.40	20			(NA,NA)	1.00	0.00	(1,1)			(NA,NA)			(NA,NA)

Table S11. Processing time and memory usage of CODEX2 for 1000 Genome Project Data. Processing time and memory usage of CODEX2 for 1000 Genome Project Data. Different number of exons are randomly selected from the 1000 Genomes Project WES data. CODEX2 is ran in the negative control region mode on a high-performance cluster. Time unit is minute (min); memory usage unit is Megabyte (Mb). Results are averaged from 5 runs.

# of exons	proc time	memory usage
2000	23.57	7.00
4000	39.61	11.51
6000	67.28	16.01
8000	76.73	20.50
10000	109.51	24.98
